# Nanoindentation Investigation of Temperature Effects on the Mechanical Properties of Nafion^®^ 117

**DOI:** 10.3390/polym8090344

**Published:** 2016-09-20

**Authors:** Re Xia, Hongjian Zhou, Runni Wu, Wen-Ping Wu

**Affiliations:** 1 School of Power and Mechanical Engineering, Wuhan University, Wuhan 430072, China; xiare@whu.edu.cn (R.X.); jackzhou@whu.edu.cn (H.Z.); nina@whu.edu.cn (R.W.); 2 Department of Engineering mechanics, School of Civil Engineering, Wuhan University, Wuhan 430072, China

**Keywords:** perfluorosulfonic acid (PFSA) membranes, Nafion^®^ 117, temperature effects, mechanical properties, nanoindentation

## Abstract

Operating temperature can be a limiting factor in reliable applications of Proton Exchange Membrane (PEM) fuel cells. Nanoindentation tests were performed on perfluorosulfonic acid (PFSA) membranes (Nafion^®^ 117) in order to study the influence of the temperature condition on their mechanical properties. The hardness and reduced modulus of Nafion^®^ 117 were measured within a certain temperature range, from 10 to 70 °C. The results indicate that both hardness and elastic modulus show non-monotonic transition with the increase of the test temperature, with reaching peak values of 0.143 and 0.833 GPa at 45 °C. It also found that the membranes have a shape memory effect and a temperature dependent shape recovery ratio.

## 1. Introduction

Perfluorosulfonic acid (PFSA) polymers, such as Nafion^®^ membranes [1], are widely used as the solid electrolyte in Proton Exchange Membrane (PEM) fuel cells and waterelectrolyzers. Today, a major concern in PFSA membranes is to continue to improve the sustainability and to extend their service life, which can be significantly reduced by the combined effects of changing chemical and mechanical environments [2]. During operation, chemical degradation and mechanical failure due to hydration and temperature cycles may be the most prominent factors restricting the durability of PEMs [3,4,5,6]. Therefore, it is very important to understand their mechanical stability changes with respect to operating conditions (humidity, temperature, etc.).

It has been found that the elastic modulus and the proportional limit stress of PFSA membranes decrease with increasing humidity and temperature, and that the change trend becomes weaker when the temperature increases to a certain value. Compared with humidity, higher temperature can bring about enhanced effects on lowering break stress and rising break strain [7,8]. The fatigue lifetime of the membranes also strongly depends on the test temperature, relative humidity and applied stress. Compared with the effect of increasing humidity, higher temperature can bring about enhanced effects on lowering break stress and raising break strain as well as reducing fatigue life [9]. However, humidity can have more complex affections on the mechanical properties of the membrane. For Nafion^®^ 117, dry samples show a lower elastic modulus than hydrated ones, with temperature ranging from 50 to 100 °C [10]. The different history of thermal treatment will cause different water uptakes and microstructures, which, in turn, may result in mechanical variation of a PFSA membrane [11]. The mechanical properties of a PFSA membrane are also affected by the deformation rate, and during a uniaxial tensile test, increasing the loading rate should strengthen the elastic modulus and the proportional limit stress, as well as the hardening modulus [12,13,14]. Then, through forming composite membranes, not only conductivity and stiffness of PFSA membranes, but also the stability of these two properties can be significantly improved [15,16]. Meanwhile, a considerable amount of simulation research and micromechanical models have been elaborated during the last decade, which also efficiently enriches the understanding of PFSA degradation and failure mechanisms [17,18,19].

In the present work, we focus on the evolution of mechanical properties of PFSA membranes with rising environmental temperature. Using the method of nanoindentation, the hardness and reduced modulus of Nafion^®^ 117 membranes were tested at temperatures between 10 and 70 °C, and the effects of indentation depth and applied temperature on the mechanical response of the membrane was investigated.

## 2. Materials and Experiments

Nafion^®^ PFSA membranes are non-reinforced films, based on Nafion^®^ PFSA polymer, a perfluorosulfonic acid/polytetrafluoroethylene (PTFE) copolymer in the acid (H^+^) form. Commercially available DuPont’s Nafion^®^ N-117 (Wilmington, DE, USA) was used in this study, with a typical thickness of 183 μm, basis weight of g/m^2^ and conductivity of 0.083 S/cm. Membranes that were used without any pretreatment.

Quasi-static nanoindentation was carried out on the samples using a nanoindenter (Triboscope TI950, Hysitron Inc., Minneapolis, MN, USA) equipped with a commercial heating stage. The heating stage consists of an upper stage component and a bottom stage component, and each of has an electrical heating element and liquid cooling system. The sample is clamped between the upper stage component and the bottom stage component. Thermocouples embedded in the upper stage and bottom stage are used for temperature feedback to maintain a constant sample temperature. The temperature control stage allows for temperatures ranging from −20 to 400 °C. A three-sided pyramidal diamond tip (Berkovich tip) with a face angle of 142.3° and a radius of curvature of ~100 nm was selected. A peak load of 100 μN was applied at a rate of 20 μN/s and then the indenter was maintained at that load for 5 s (hold-at-the-peak method), followed by unloading at a constant speed for 5 s. In order to investigate the temperature effects on the mechanical properties of Nafion^®^ 117, the load temperature in the test chamber was set at a range of 10 to 70 °C, with an increment value of 10 °C. The temperature was increased to the set point at a rate of 2 °C/min, and then allowed to stabilize for an hour, keeping both the tip and samples at the same temperature during the nanoindentation tests. Environment humidity was at a relative humidity of 58%. A minimum of 9 indents were collected for each sample at each temperature point.

Hardness, *H* and elastic modulus (reduced modulus *E*_r_) were calculated from the load-displacement data obtained by nanoindentation, based on the Oliver-Pharr method [20]. The elastic modulus of membranes is related to the reduced modulus by:

(1) $$\frac{1}{E_{r}}=\frac{\left(1-\nu^{2}\right)}{E}+\frac{\left(1-\nu_{i}^{2}\right)}{E_{i}}$$

where *E* and *ν* are the elastic modulus and Poisson’s ratio of the material, respectively, and *E*_i_ and *ν*_i_ those of the indenter. Then, the elastic modulus of membrane was calculated by using *ν* = 0.5 for Nafion^®^ 117 and *E*_i_ = 1140 GPa, *ν*_i_ = 0.07 for the diamond indenter. However, for low modulus polymers (*ν = 0.3~0.5*), the correction is lower than 0.1% for the second term and the first one only modified the absolute values, resulting in the insignificant difference between *E*_r_ and *E.*

## 3. Results and Discussion

**Figure 1:**
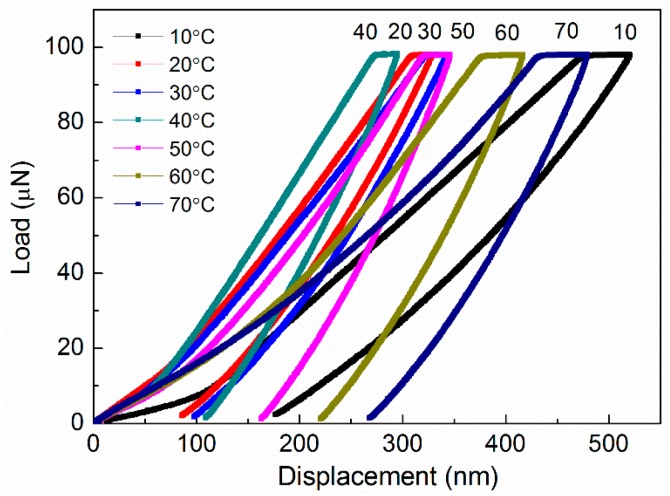
Comparison of load vs. displacement curves of Nafion^®^ 117 membrane at different temperatures, from 10 to 70 °C.

Figure 1 shows representative indentation load-depth curves (*P*–*h* curves) of Nafion^®^ 117 membranes at different temperatures, *T*, with the same peak load of 100 μN. Nanoindentation curves shifted to the left, and maximum depth reduced due to increasing hardness. The horizontal segment at the peak load represents that the indenter continues to penetrate into the membranes, giving an indication of the result of creep, even at 10 °C. If weakening the effect of creep is desired, a long hold period, as well as a fast indenter unloading rate, are required [21,22]. Through *P*–*h* curves comparison, one can find that the maximum indentation depths and residual depths show obvious differences with varying temperature conditions. Although all curves reveal that the maximum penetration depth occurred at *T* = 10 °C and the maximum residual depth at *T* = 70 °C, both the lowest penetration depth and residual depth were observed at *T* = 40 °C.

**Figure 2:**
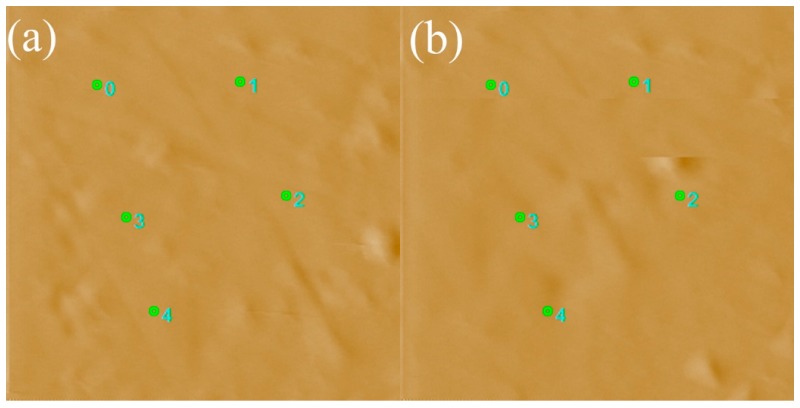
Indentation surface of the membrane (**a**) before and (**b**) after test at 20 °C. Numbers 0–4 represent the marking-points of five indentations.

**Figure 3:**
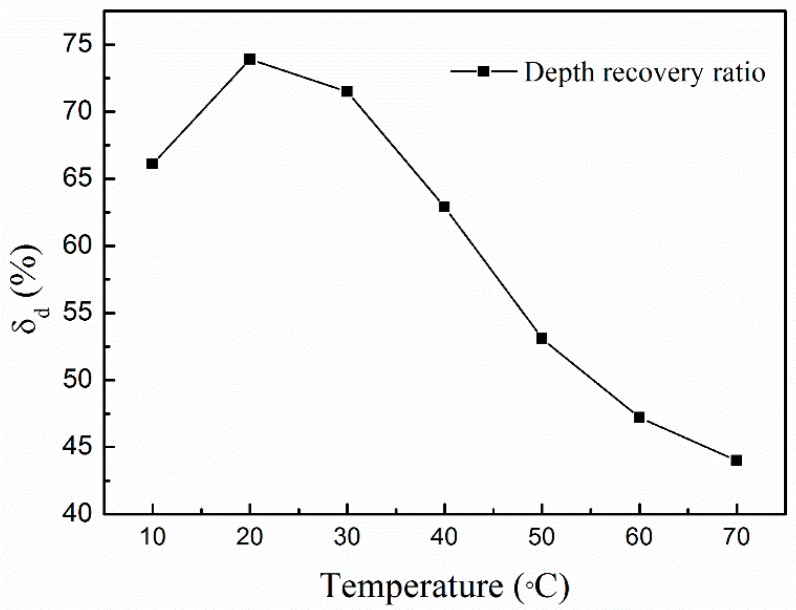
Variation in depth recovery ratio with elevated temperatures.

It can be seen in Figure 1 that there is an evident recovery effect of the indents in the depth direction, which most commonly occur in a nanoindentation test of shape memory alloys (SMA). Then, by comparing the indentation surface in the membrane before and after test (at 20 °C), it confirms that there is no apparent residue of indentations, as shown in Figure 2. Similarly, Nafion nanofibers were found to exhibit excellent shape memory performance when deformed and recovered above the transition temperature, having shape recovery ratios above 90% [23]. The degree of indent recovery in the depth direction can be determined quantitatively by defining a recovery ratio [24,25,26]:

(2) $$\delta_{d}=\frac{\delta_{\max}-\delta_{r}}{\delta_{\max}}$$

where the depth recovery ratio $\delta_{d}$ is a measure of recoverable work done during indentation, $\delta_{r}$ is the remnant depth at unloading, and $\delta_{\max}$ is the depth at the maximum indentation load. $\delta_{d}$ approaches zero when there is no recovery (i.e., indentation is equal to maximum penetration), while it is 1 when the elastic recovery is complete. All data related to the recovery ratio are listed in Table 1 and the variations in indent recovery ratios at elevated temperatures are shown in Figure 3.

It can be seen that the recovery behaviors of membranes differentiate significantly when the test temperature varies. At 20–30 °C (about room temperature), the membrane has a larger recovery during unloading and the maximum ratio, 73.9%, occurs at 20 °C. With an increase of temperature, the recovery gradually decreases (except at 10 °C), and then a relatively small proportion of recovery occurred at 70 °C, as shown in Figure 3. Temperature-dependent recovery ability may result from the change in phase state of the membranes. It is well known that the shape memory effect in SMA is the result of thermoelastic martensitic transformation from the cubic (B2) to the monoclinic (B19′) structure [27,28,29]. However, in polymers, Fulcher et al. [30] suggest that the shape recovery ability may arise from a rearrangement of long molecular chains, so that new bonds are formed and a fixed shape is held. They emphasized that the best method to characterize the shape memory effect is to activate the rubbery state of the polymer at an elevated deformation temperature above the glass transition point.

**Figure 4:**
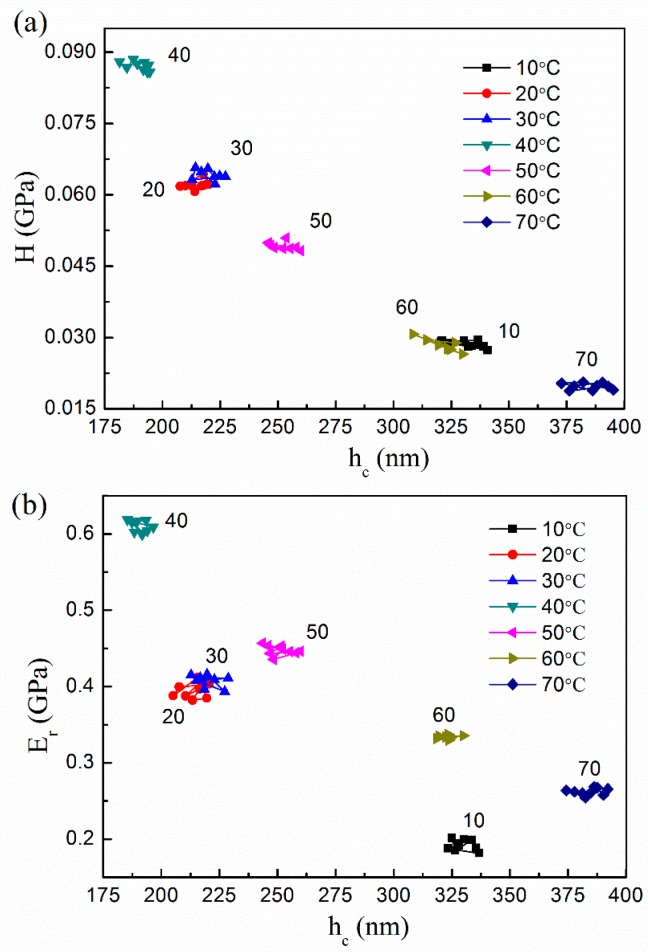
Variation of (**a**) hardness and (**b**) reduced modulus of membranes along the contact depth at different temperatures.

Figure 4 shows the relationship between hardness/reduced modulus with contact depth *h*_c_ for different values of the test temperatures. Clearly, the indenter contact depth at a peak load 100 μN does not have a monotonic relation with rising temperature, and comes to a turning point where the deformation temperature reaches 40 °C. At 40 °C, depth is lowered to a minimum of about 180 nm. Below this point, a rise in temperature decreases the contact depth, with a higher value of about 330 nm at 10 °C. In the range of 40 to 70 °C, depth increases with increasing temperature, reaching a maximum value of about 390 nm at 70 °C.

**Figure 5:**
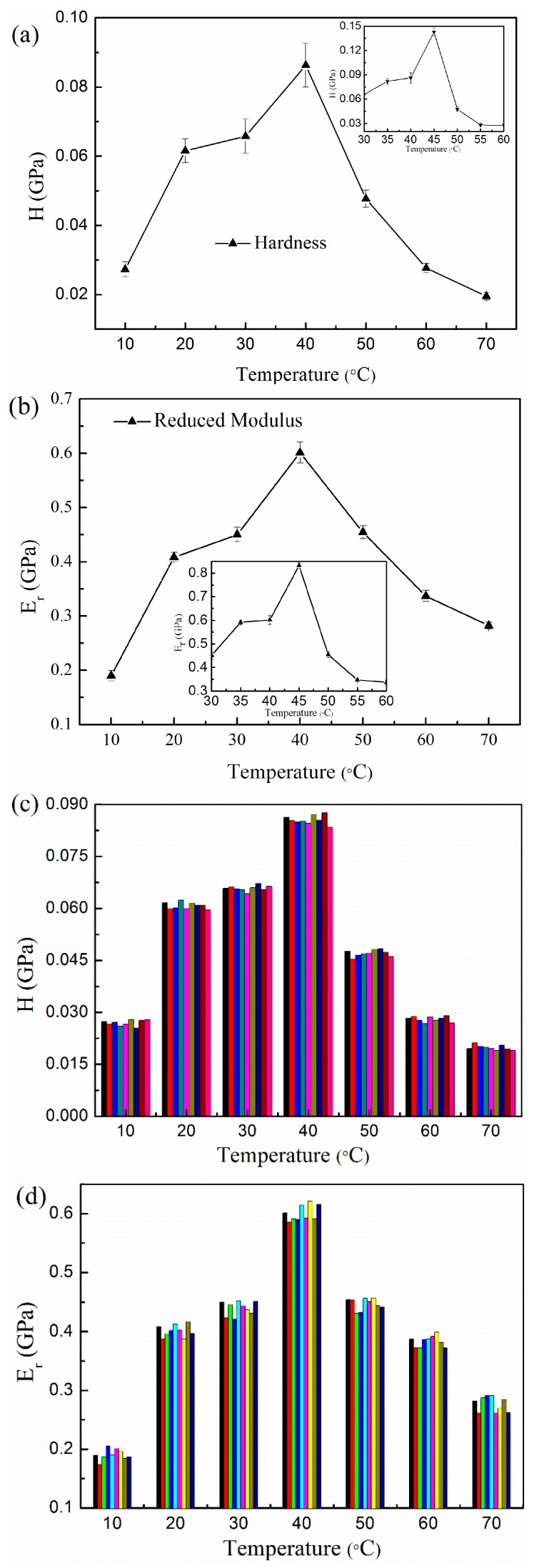
Effect of elevated temperatures on (**a**) average hardness and (**b**) reduced modulus of membranes (insets are the added measurements at 35, 45 and 55 °C), and the typical distribution of measured (**c**) hardness and (**d**) reduced modulus at different temperatures. Different colors in (**c**) and (**d**) correspond to different measure points.

Hardness and reduced modulus, as function of various temperatures, are depicted in Figure 5. It can be seen that they represent a similar variation tendency with elevated temperatures, as shown in Figure 4. The critical point of the transition also occurs at 40 °C, in accordance with that of penetration depth. In this situation, a higher hardness and reduced modulus are obtained, at values close to 0.088 and 0.62 GPa. Both properties are strengthened when the temperature rises from 10 to 40 °C, and then weaken when the test temperature is increased further. The difference is that the lowest hardness occurs at the highest temperature of 70 °C, with an approximate value of 0.025 GPa, whereas the reduced modulus reaches a minimum at 10 °C with 0.27 GPa. Compared with the hardness, the measured modulus is likely to have a larger discrepancy due to polymers creep. Only using an elastic contact mechanics model to analyze the indenter results of viscoelastic polymers usually leads to an overestimation in elastic modulus [31]. To confirm the transition of Nafion properties, we conducted extra three group tests under the temperature conditions near the peak value zone (35, 45 and 55 °C). Clearly, the results further prove similar changing trends of hardness and reduced modulus, as shown in the insets of Figure 5a,b. Then, the corresponding peaks move to the position at 45 °C, with values of 0.143 and 0.833 GPa, respectively. Moreover, we attempted to test the membranes at higher temperatures (over 80 °C), but the disparity of the nanoindentation data became too wide, especially near 100 °C. This is likely a result of glass transition, since Nafion 117 membranes are known to have a glass transition temperature range of 120–140 °C.

One possible reason for the transition (at 45 °C) is that the measurements presents a combined effect of membrane dehydration followed by a softening effect with increasing temperature. The mechanical response of Nafion membranes are strongly influenced by both relative humidity and temperature, but during nanoindentation tests, it is hard to control the relative humidity when varying the temperature. The heating chamber has an initial relative humidity (RH) of 58% (environment humidity), and was isolated from environmental condition during heating. Therefore, the membrane water content is not constant, and the influences of moisture and temperature could be added together. To get a better application of nanoindentation in PFSA membrane tests, developing a combined temperature and humidity controlling system would be a technical challenge. 

Although the nanoindentation technique is proven to be suitable for determining the mechanical properties of polymers, it encounters certain unsolved difficulties. The applicability of the Oliver-Pharr method has been discussed and modified by Cheng et al. and Nagn et al. for viscoelastic solids [32,33,34,35]. In addition, Díez-Pascual indicates that creep influences the condition of pure elastic unloading, even in the case of long holding times and high unloading rates, and is still a matter of concern; choosing which criterion to fit the unloading data to is also deserving of attention [21]. It is well known that long holding times and high unloading rates can be used to minimize the creep effect during nanoindentation tests, though the appropriate experimental conditions may be different for different material properties. Therefore, it is necessary to perform future research on the optimization criteria of loading/unloading rates and holding periods for Nafion membranes. Furthermore, due to the creep effect of viscoelastic solids, it is not ideal to describe the instrumented-indentation measures of time-dependent modulus in terms of “elastic modulus” and “reduced modulus”. Rather, it should be the “instantaneous” modulus of viscoelastic solids (*E*_0_), which was suggested by Cheng et al. [36] and also used by Oyen [37]. Future work will focus on these issues.

## 4. Conclusions

In this work, the mechanical properties of Nafion^®^ 117 membranes were examined for a range of temperature conditions using a nanoindenter equipped with a hot-stage heating system. The load-depth curves of the membranes were obtained at various temperatures, all of which suggest that they may exhibit temperature-dependent shape recovery ratios and a noticeable shape memory effect, even at 10 °C. The hardness and reduced modulus of the membranes do not vary monotonously with increasing temperature, and, instead, present the tendency of increasing first and then decreasing, when the temperature increases from 10 to 70 °C, reaching their maximum values of 0.143 and 0.833 GPa at 45 °C.

## Figures and Tables

**Table 1:** Variation in recovery ratio of Nafion^®^ 117 membranes with different temperatures.

Temperature (°C)	10	20	30	40	50	60	70
Depth at peak load, $\delta_{\max}$ (nm)	520.5 ± 11.6	328.0 ± 9.4	342.2 ± 9.4	294.2 ± 6.4	346.2 ± 8	416.5 ± 6.4	479.0 ± 9.4
Remnant depth, $\delta_{r}$ (nm)	176.4 ± 9.5	85.5 ± 5.1	97.4 ± 6.9	109.0 ± 4.5	162.5 ± 3.7	220.0 ± 5.7	267.5 ± 12
Depth recovery ratio, $\delta_{d}$	66.1%	73.9%	71.5%	62.9%	53.1%	47.2%	44%
